# The performance of sequence symmetry analysis as a tool for post-market surveillance of newly marketed medicines: a simulation study

**DOI:** 10.1186/1471-2288-14-66

**Published:** 2014-05-15

**Authors:** Nicole L Pratt, Jenni Ilomäki, Chris Raymond, Elizabeth E Roughead

**Affiliations:** 1 Quality Use of Medicines and Pharmacy Research Centre, Sansom Institute, School of Pharmacy and Medical Sciences, University of South Australia, Adelaide, SA, Australia; 2 Department of Health and Ageing, Pharmaceutical Evaluation - DUSC/ESC Section, Pharmaceutical Evaluation Branch, Pharmaceutical Benefits Division, Canberra, ACT, Australia

## Abstract

**Background:**

Sequence symmetry analysis (SSA) is a potential tool for rapid detection of adverse drug events (ADRs) associated with newly marketed medicines utilizing computerized claims data. SSA is robust to patient specific confounders but it is sensitive to the underlying utilization trends in the medicines of interest. Methods to adjust for utilisation trends have been developed, however, there has been no systematic investigation to assess the performance of SSA when variable prescribing trends occur. The objective of this study was to evaluate the validity of SSA as a signal detection tool for newly marketed medicines.

**Methods:**

Randomly simulated prescription supplies for a population of 1 million were generated for two medicines, DrugA (medicine of interest) and DrugB (medicine indicative of an adverse event). Scenarios were created by varying medicine utilization trends for a newly marketed medicine (DrugA). In addition, the magnitude of association between DrugA and DrugB was varied. For each scenario 1000 simulations were generated. Average Adjusted Sequence Ratios (ASR), bootstrapped 95% confidence intervals (CIs), percentage of CI's which covered the expected ASR and percent relative bias were calculated.

**Results:**

When no association was simulated between DrugA and DrugB, over 95% of SSA CI's covered the expected ASR (ASR = 1) and relative bias was 1% or less irrespective of medicine utilization trends. In scenarios where DrugA and DrugB were associated (ASR = 2), unadjusted SR's were underestimated by between 11.7 and 15.3%. After adjustment for trend, ASR estimates were close to expected with relative bias less than 1%. Power was over 80% in all scenarios except for one scenario in which medicine uptake was gradual and the effect of interest was weak (ASR = 1.2).

**Conclusions:**

Adjustment for underlying medicine utilization patterns effectively overcomes potential under-ascertainment bias in SSA analyses. SSA may be effectively applied as a safety signal detection tool for newly marketed medicines where sufficiently large health claim data are available.

## Background

Sequence Symmetry Analysis (SSA) has been suggested as a tool to complement current systems of post-marketing surveillance of medicines which use spontaneous reporting databases [1]. The method, developed by Hallas [2], has been used increasingly with administrative claims data to investigate adverse effects of medicines including ace-inhibitor induced cough [3,4], inhaled corticosteroid induced oral candidiasis [5], non-steroidal anti-inflammatory induced stroke [6], and isotretinoin and cardiovascular medicine induced depression [2,7]. A validation study, using known adverse drug reactions from randomized controlled trials as the gold standard, demonstrated that SSA has high sensitivity and moderate specificity for detecting safety signals [1], and had similar sensitivity and specificity to signal detection methods employed in spontaneous reporting databases. An advantage of the SSA method is its ease of application, computational speed and minimal dataset requirements. The method utilizes existing health claims datasets and due to the within person study design does not require numerical adjustment for time invariant patient specific confounders.

Sequence Symmetry Analysis assesses the association between two medicines in prescription claims data by comparing the sequence of initiation of each medicine during the study period or within a specified period of time for an individual. One of the medicines (DrugA) is the exposure medicine of interest and the other medicine (DrugB) indicates a possible adverse event for which a medicine may have been prescribed. In practice, SSA works by determining the first use of DrugA for an individual (ie the first supply date for DrugA in the available dataset for each individual). The same is done for DrugB. Then for each individual, DrugA and DrugB initiations within a defined period of time, for example 12 months, are selected and included in the analysis [8]. The ratio of the number of persons with DrugB initiated after DrugA is compared to the number of persons with DrugB initiated before DrugA. This ratio is described as the crude sequence ratio (CSR). If there is no association between the medicines the CSR will be approximately unity. If there is asymmetry in the distribution of initiation of DrugB after DrugA compared to before DrugA, it may imply an association between the medicines. The sequence ratio is an estimate the incidence rate ratio of the outcome event in exposed compared to non-exposed person time [2]. The within person study design ensures that the analysis is robust towards patient specific confounders that are stable over time, however, the analysis is sensitive to prescribing trends over time [2]. For example, if DrugA is decreasing in use but there is no trend in the utilization of DrugB over time it is likely that there will be more people starting DrugB after DrugA just by chance, even if the medicines are not associated. This may lead to an incorrect positive association between the medicines. Alternatively, an increase in DrugA over time will create an excess of patients with DrugA prescribed second and therefore an underestimate the association between DrugA and DrugB if one really exists. This latter scenario may happen, for example, when a new medicine enters the market. The implication of not allowing for prescribing trends would likely be an underestimate of a potential adverse event of a medicine. In order to adjust for such temporal trends, a null-effect sequence ratio is calculated. The null-effect sequence ratio is the sequence ratio that would be expected in the absence of a causal association, given the trend in incident medicine use in the background population [8]. The crude sequence ratio is adjusted for temporal trends in medicine use over time by dividing by the calculated null-effect sequence ratio.

While the validity of SSA has been assessed using known adverse drug reactions as the gold standard [1], the validity of the method has not been assessed systematically using simulations studies where a known association is injected into the study population under varying scenarios of medicine utilisation. The advantage of simulation studies is that the validity of the sequence ratio can be determined in the presence of varying trends in incident medicine use and the validity of the null-effect sequence ratio in adjusting for temporal trends in prescribing can be assessed.

The aim of this study was to evaluate the performance of SSA in scenarios which varied 1) the magnitude of the associations between the medicines using simulated prescription orders and 2) the trend in incident medicine use of a medicine new to market.

## Methods

Sequence symmetry analysis was used to examine the association between DrugA and DrugB. The crude sequence ratio (CSR) was calculated as the ratio of the number of persons with DrugB after DrugA to the number of persons with the DrugB before DrugA. The null-effect sequence ratio (NSR) was calculated using the method described in Tsiropolous [9]. The adjusted sequence ratio (ASR) was calculated as the crude sequence ratio divided by the null-effect sequence ratio. We performed SSA on 1000 simulations of each of the scenarios. Average ASR with bootstrapped 95% confidence intervals (CI) (500 replicates), the proportion of simulations in which 95% CI included the expected ASR (coverage probability), the proportion of simulations in which 95% CI did not include 1.00 (power) and average relative bias (difference between the estimated sequence ratio and the expected sequence ratio) were calculated.

### Simulated data

For a population of 1 million we randomly simulated prescription supplies independently for two medicines, DrugA and DrugB. For the population of 1 million, 20% of patients were randomly selected to initiate drugA and 20% of patients to initiate DrugB during the study period. This meant that by chance 4% of patients would be initiated on both DrugA and DrugB. Since medicine supply dates were randomly generated, the number of patients prescribed DrugA then DrugB should be similar to the number of patients who were prescribed DrugB then DrugA. In this scenario the expected sequence ratio would be 1.0, that is, no asymmetry in prescription supplies.

**Figure 1:**
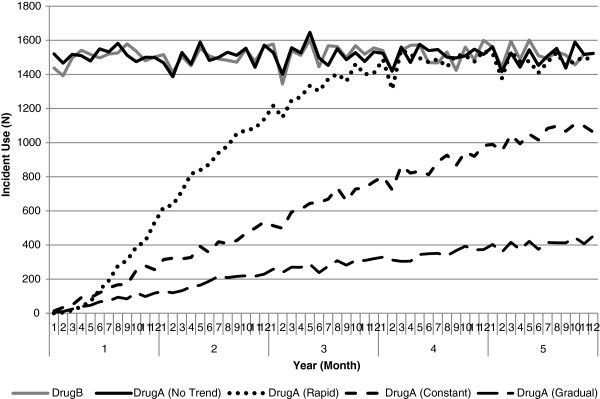
Simulated trends for incident use of DrugA and DrugB.

In order to generate the trend in medicine utilization, DrugA supply dates were randomly generated using the following distribution functions; uniform distribution for no trend, the log-normal distribution for a rapidly increasing trend, weibull distribution for a constant increasing trend and gamma distribution to simulate a gradual increasing trend in DrugA (Figure 1). In all scenarios DrugB supply dates were randomly generated using the uniform distribution.

We also simulated six scenarios in which the magnitude of the associations between DrugA and DrugB were varied. In the first scenario, there was no association between the medicines and the expected sequence ratio was 1.00. In the other scenarios the expected sequence ratios were 0.6, 0.8, 1.20, 1.50, 2.00 and 3.00. The associations were induced by forcing a percentage of patients who were not initiated on either medicine to be allocated a DrugA then a DrugB supply, in that order. SSA was restricted to sequences of medicine initiations within 12 months of each other as, when used in practice, this is likely to limit the effect of age and other potential time-varying covariates on the probability of exposure and outcome. Sensitivity analyses were performed in which we reduced the prevalence of medicine use to 5%.

SAS Version 9.4 was used for all simulations and analyses.

## Results

Table 1 presents the simulation results of each medicine utilization scenario for the different effect sizes.

**Table 1:** Sequence symmetry results of the 1000 simulations for different scenarios of medicine utilisation trends and effect sizes, for a population size of 1 million and 20% population prevalence of use of each drug

			**Crude sequence ratio**				**Adjusted sequence ratio**			
**Medicine utilisation scenario**	**Pairs (N)**	**Null SR**	**Crude SR (95% CI)**	**Cover-age (%)**	Power* (%)	**Relative bias (%)**	**Adjusted SR (95% CI)**	**Cover-age (%)**	Power* (%)	**Relative bias (%)**
**Expected ASR = 1.0**										
***No trend***	3597	1.00	1.00 (0.94-1.07)	95.5	2.2	0.38	1.00 (0.94-1.07)	95.6	2.1	0.38
***Gradual***	656	0.85	0.86 (0.73-1.00)	47.6	0.0	−13.85	1.00 (0.85-1.17)	94.4	2.1	1.02
***Constant***	1604	0.84	0.84 (0.76-0.93)	7.3	0.0	−15.34	1.00 (0.90-1.11)	95.2	1.9	0.46
***Rapid***	1811	0.88	0.88 (0.80-0.97)	22.4	0.0	−11.71	1.00 (0.91-1.10)	96.1	2.2	0.46
**Expected ASR = 1.2**										
***No trend***	3956	1.00	1.20 (1.13-1.28)	94.2	100.0	0.35	1.20 (1.13-1.28)	94.7	100.0	0.36
***Gradual***	717	0.85	1.03 (0.88-1.19)	42.8	5.1.0	−13.51	1.21 (1.03-1.40)	94.6	66.4	1.43
***Constant***	1754	0.84	1.01 (0.92-1.11)	4.8	2.9	−15.52	1.20 (1.09-1.32)	94.8	96.0	0.23
***Rapid***	1983	0.88	1.05 (0.96-1.15)	18.9	19.8	−11.85	1.20 (1.09-1.31)	94.2	96.8	0.31
**Expected ASR = 1.5**										
***No trend***	4498	1.00	1.50 (1.41-1.59)	95.2	100.0	0.19	1.50 (1.41-1.59)	95.6	100.0	0.18
***Gradual***	806	0.85	1.29 (1.11-1.48)	41.0	92.2	−13.82	1.51 (1.30-1.73)	95.6	100.0	1.07
***Constant***	1973	0.84	1.27 (1.15-1.39)	3.9	100.0	−15.29	1.50 (1.37-1.65)	96.0	100.0	0.50
***Rapid***	2238	0.88	1.32 (1.21-1.44)	16.6	100.0	−11.67	1.50 (1.38-1.64)	95.6	100.0	0.50
**Expected ASR = 2.0**										
***No trend***	5395	1.00	2.00 (1.90-2.12)	94.2	100.0	0.42	2.00 (1.90-2.12)	94.7	100.0	0.42
***Gradual***	957	0.85	1.72 (1.49-1.96)	36.7	100.0	−13.92	2.01 (1.74-2.29)	95.2	100.0	0.95
***Constant***	2338	0.84	1.69 (1.55-1.84)	2.4	100.0	−15.29	2.00 (1.83-2.19)	95.7	100.0	0.50
***Rapid***	2660	0.88	1.76 (1.62-1.91)	11.2	100.0	−11.69	2.00 (1.85-2.17)	96.1	100.0	0.48
**Expected ASR = 3.0**										
***No trend***	7197	1.00	3.00 (2.85-3.17)	95.1	100.0	0.26	3.00 (2.85-3.17)	95.1	100.0	0.27
***Gradual***	1260	0.85	2.57 (2.26-2.90)	29.2	100.0	−13.92	3.01 (2.65-3.41)	95.8	100.0	0.98
***Constant***	3074	0.84	2.54 (2.35-2.75)	0.9	100.0	−15.00	3.01 (2.79-3.26)	95.4	100.0	0.82
***Rapid***	3510	0.88	2.63 (2.45-2.84)	8.6	100.0	−11.72	3.00 (2.79-3.24)	93.5	100.0	0.44
**Expected ASR = 0.6**										
***No trend***	4799	1.00	0.60 (0.56-0.64)	94.0	100.0	−0.03	0.60 (0.56-0.64)	94.0	100.0	−0.03
***Gradual***	893	0.85	0.51 (0.44-0.58)	32.6	100.0	−14.61	0.60 (0.52-0.68)	95.7	100.0	0.13
***Constant***	2187	0.84	0.51 (0.46-0.55)	23	100.0	−15.54	0.60 (0.55-0.66)	96.3	100.0	0.21
***Rapid***	2456	0.88	0.53 (0.48-0.57)	12.1	100.0	−11.90	0.60 (0.55-0.65)	96.3	100.0	0.24
**Expected ASR =0.80**										
***No trend***	4048	1.00	0.80 (0.75-0.85)	95.7	100.0	0.11	0.80 (0.75-0.85)	95.7	100.0	0.10
***Gradual***	745	0.85	0.68 (0.58-0.79)	39.4	100.0	−14.30	0.80 (0.68-0.92)	95.5	86.3	0.51
***Constant***	1823	0.84	0.68 (0.61-0.74)	4.8	100.0	−15.49	0.80 (0.73-0.88)	95.0	99.9	0.27
***Rapid***	2054	0.88	0.70 (0.64-0.77)	15.8	100.0	−12.17	0.80 (0.73-0.87)	95.6	99.9	−0.07

*Note: When the expected Sequence ratio =1, Power is the type I error.

### Expected ASR = 1.00

When the expected sequence ratio was 1.0, corresponding to no asymmetry in the order of prescriptions supplied, Type 1 error was identified in less than 2.2% of simulations for all medicine utilization scenarios. When no trend was simulated the crude and adjusted sequence ratios were similar and relative bias was 0.38%. The crude sequence ratios were underestimated by 13.9%, 15.3% and 11.7% when the simulated trend was gradual, constant and rapid respectively. After adjustment for trend using the null sequence ratio relative bias was reduced to 1% or less.

### Expected ASR = 1.2

In the scenario where there was no trend in medicine utilization, 94.7% of CI’s covered the expected ASR (ASR = 1.2) and power was 100%. When DrugA utilization was varied the CSR’s were under-estimated by 11.9% to 15.5%. After adjustment for trend, the mean ASR was 1.2, with over 94% of CIs containing the expected sequence ratio. In the case of a gradually increasing trend, power was only 66.4% compared to 96.0% and 96.8% when there was a constant or rapid increasing trend respectively. Relative bias was reduced to less than 1.5% in all scenarios after adjustment for trend.

### Expected ASR = 1.5, 2.0 and 3.0

When there was no trend in medicine utilization, approximately 95% of CI’s covered the expected ASR and power was 100%. When DrugA was simulated to increase over time ASRs were close to the expected with relative bias 1% or less.

### Expected ASR = 0.8 and 0.6

When there was no trend in medicine utilization and the expected ASR was 0.6, 94% of CI’s covered the expected ASR and power was 100%. When DrugA was simulated to increase over time ASRs were close to expected with relative bias 0.13 to 0.24%. Power was 86.3% when the expected ASR was 0.8 and the uptake in DrugA was gradual. Power was 99.9% for all other scenarios where the expected ASR was 0.8.

### Sensitivity analyses variation in medicine utilization prevalence

The results of the analysis in which the population proportion of drug use was changed to 5% is summarised in Table 2. When the expected SR was 1.0, type 1 error was between 0.1 and 1.6% and relative bias was between 2.4 and 12.2%. For an expected ASR of 1.2, power was 20.8% when there was no trend in A, 8% when there was a constant or rapid trend and relative bias was between 2.8% and 12.9%. For effect sizes 1.5, 2.0 and 3.0 when no trend was simulated, power was over 85%. For an effect size of 1.5, insufficient power was found for all trends. For an effect size of 2.0, power was less than 80% only for a gradual trend in DrugA.

**Table 2:** Sequence symmetry results of the 1000 simulations for different scenarios of medicine utilisation trends and different effect sizes, for a population size of 1 million and 5% incidence of use of each drug in the population

			**Crude sequence ratio**				**Adjusted sequence ratio**			
**Medicine utilisation scenario**	**Pairs (N)**	**Null SR**	**Crude SR (95% CI)**	**Cover-age (%)**	Power* (%)	**Relative bias (%)**	**Adjusted SR (95% CI)**	**Cover-age (%)**	Power* (%)	**Relative bias (%)**
**Expected ASR = 1.0**										
***No trend***	225	1.00	1.01 (0.75-1.30)	94.3	1.8	2.42	1.01 (0.75-1.30)	94.5	1.6	2.42
***Gradual***	41	0.85	0.90 (0.31-1.60)	86.8	0.0	−4.33	1.06 (0.37-1.88)	94.5	0.1	12.18
***Constant***	100	0.84	0.85 (0.52-1.23)	81.1	0.1	−12.58	1.01 (0.61-1.46)	94.7	0.2	3.73
***Rapid***	113	0.88	0.90 (0.57-1.27)	87.3	0.0	−7.75	1.03 (0.65-1.45)	95.2	1.3	4.97
**Expected ASR = 1.2**										
***No trend***	247	1.00	1.21 (0.91-1.55)	95.9	20.7	2.84	1.21 (0.91-1.55)	95.7	20.8	2.84
***Gradual***	44	0.85	1.08 (0.40-1.91)	90.6	0.0	−3.73	1.27 (0.47-2.24)	96.9	0.5	12.90
***Constant***	110	0.84	1.04 (0.65-1.47)	81.5	1.3	−11.45	1.23 (0.77-1.75)	94.9	7.9	5.05
***Rapid***	124	0.88	1.07 (0.70-1.49)	87.4	1.4	−8.70	1.22 (0.79-1.70)	95.8	7.8	3.90
**Expected ASR = 1.5**										
***No trend***	280	1.00	1.51 (1.15-1.92)	95.6	85.5	2.54	1.51 (1.15-1.92)	95.4	85.8	2.52
***Gradual***	50	0.85	1.36 (0.56-2.33)	86.7	0.6	−3.58	1.59 (0.66-2.74)	95.6	5.6	13.10
***Constant***	124	0.84	1.29 (0.84-1.82)	81.1	13.3	−11.39	1.54 (1.00-2.16)	96.2	45.4	5.12
***Rapid***	139	0.88	1.35 (0.90-1.87)	86.9	24.6	−7.53	1.54 (1.03-2.13)	95.9	52.3	5.20
**Expected ASR = 2.0**										
***No trend***	337	1.00	2.03 (1.57-2.55)	96.1	100.0	2.91	2.03 (1.57-2.55)	96.2	100.0	2.89
***Gradual***	60	0.85	1.79 (0.81-3.01)	87.7	17.7	−4.59	2.10 (0.95-3.53)	95.6	40.6	11.87
***Constant***	146	0.84	1.72 (1.15-2.39)	80.6	76.1	−11.44	2.04 (1.37-2.84)	96.1	97.0	5.08
***Rapid***	166	0.88	1.79 (1.23-2.45)	87.5	90.7	−7.96	2.04 (1.40-2.79)	97.0	98.7	4.71
**Expected ASR = 3.0**										
***No trend***	450	1.00	3.03 (2.40-3.77)	96.4	100.0	2.84	3.03 (2.40-3.77)	96.2	100.0	2.85
***Gradual***	78	0.85	2.67 (1.30-4.47)	87.8	90.8	−3.94	3.14 (1.52-5.24)	96.3	97.9	12.66
***Constant***	192	0.84	2.59 (1.79-3.55)	80.5	100.0	−11.16	3.07 (2.12-4.21)	97.5	100.0	5.39
***Rapid***	219	0.88	2.67 (1.90-3.59)	83.4	100.0	−8.50	3.03 (2.16-4.09)	96.0	100.0	4.12
**Expected ASR = 0.6**										
***No trend***	300	1.00	0.61 (0.46-0.76)	95.6	99.0	1.66	0.61 (0.46-0.76)	95.6	99.0	1.68
***Gradual***	56	0.85	0.53 (0.23-0.85)	86.8	77.9	−9.71	0.62 (0.27-1.00)	95.2	57.0	5.92
***Constant***	136	0.84	0.51 (0.33-0.70)	79.2	98.2	−14.48	0.61 (0.39-0.83)	94.9	90.0	1.46
***Rapid***	154	0.88	0.53 (0.35-0.71)	83.7	98.6	−11.09	0.60 (0.40-0.81)	94.5	91.1	1.14
**Expected ASR = 0.8**										
***No trend***	254	1.00	0.81 (0.61-1.03)	95.4	44.5	2.09	0.81 (0.61-1.03)	95.7	44.5	2.10
***Gradual***	46	0.85	0.72 (0.28-1.21)	89.1	32.7	−6.68	0.84 (0.33-1.42)	95.9	16.6	9.43
***Constant***	114	0.84	0.69 (0.43-0.97)	79.5	60.4	−13.09	0.82 (0.50-1.15)	95.0	29.1	3.14
***Rapid***	129	0.88	0.71 (0.46-0.98)	83.2	55.8	−9.95	0.81 (0.52-1.12)	94.1	30.9	2.47

*Note: When the expected Sequence ratio =1, Power is the type I error.

## Discussion

We tested SSA in different realistic scenarios for the underlying trend in uptake of a newly marketed medicine over time [9] for different effect sizes and a fixed population size of 1 million people. In all simulations adjustment for trends in prescribing using the null-effect sequence ratio appeared to effectively overcome under-ascertainment bias. When trends in medicine use were present, the crude sequence ratio underestimated the true association by 12-16%. After adjustment for underlying medicine utilisation patterns relative bias was 1 to 2%.

SSA analyses had high statistical power in all simulations with effect sizes greater than or equal to 1.5 regardless of the medicine uptake trend. For an effect size of 1.2 and a gradual uptake of the medicine, SSA had only 66% power. This is most likely a consequence of smaller numbers of patients available for analysis in the early years of a gradual medicine uptake rate. However, under the gradual medicine uptake utilization scenario, effect sizes of 1.5 and 2.0 both had nearly 100% power. Estimates of relative bias, however, were largely unaffected by sample size. These results suggest that SSA analyses may be a reliable method to identify adverse events associated with a newly marketed medicine in a sufficiently large population, particularly if the uptake of the medicine is rapid.

In all simulations coverage probability was high as was the power. Coverage and power are measures that are dependent on the sample size. In a sensitivity analysis the impact of the prevalence of the medicine use on the performance of PSSA was explored by reducing the percentage of patients initiating the medicines to 5%. In general, the lower use of medicines reduced the power of SSA but increased the coverage probability marginally. This is most likely due to the increased variability and hence the width of the confidence intervals. The performance of SSA in terms of estimating the true estimated effect was slightly affected, as relative bias of the estimates increased marginally. These results suggest that the power calculations are dependent on the percentage of use and consequently the number of pairs generated.

In practice, SSA has been shown to be robust to varying utilization patterns of medicine use [10]. An application of SSA to the investigation of the association between antipsychotics and hyperglycaemia across six countries found a consistent positive association despite varying patterns of utilization in the different populations. The significance of the association was dependent on the number of pairs generated in each country [10]. In this study we have explored how varying trends in medicine utilization may impact on the validity of SSA, however, there may be other biases to consider when implementing SSA in practice including confounding by contra-indication and protopathic bias [2].

In this simulation study we fixed the population size at 1 million and only considered a limited number of trends of medicine uptake in DrugA. In this analysis we only varied DrugA, however, in practice trends may occur in either or both DrugA and DrugB. Additionally, we only simulated scenarios where no association between DrugA and DrugB were present and where associations exist from 20% increased risk up to a tripling of risk and negative associations where the expected ASR was 0.6 or 0.8. Future work will be required to determine the validity of SSA under conditions different to those explored here, such as varying population sizes, rates of initiation of both DrugA and DrugB and for more extreme associations. In particular, further studies will be required to determine the performance of SSA in situations where very rare but serious adverse events may be expected. In this simulation we have employed the method of adjustment as described by Tsiropolous [8]. This method is an amendment from the original technique first described by Hallas [2]. The rationale for this amendment was described in the paper by Tsiropolous to account for limited time intervals allowed between the exposure medicine of interest and the adverse drug event. This is relevant to the situation simulated in this analysis in which acute adverse events are of interest. In general the method of adjustment is dependent on the situation at hand and it may be more reasonable to use the method as originally described by Hallas. In a sensitivity analysis, the method as described by Hallas [2] was implemented and similar results were found (data not shown).

A limitation of the SSA approach in practice is that it can only be applied to post-market surveillance issues where medicines are prescribed to treat the adverse event or where the outcome of interest may be identified as an admission to hospital. Examples of studies which have investigated medicine initiation as a proxy for an adverse medicine event include, insulin initiation as a proxy for acute hyperglycaemia associated with olanzapine initiation [10], and antitussive medicine initiation as a proxy for cough associated with ACE inhibitors [3,4]. Results of SSA, like all observational study designs, must be interpreted with appropriate consideration given to the sensitivity and specificity of the proxy medicine or adverse event hospital diagnosis as a measure of the adverse event of interest.

## Conclusions

The results of this simulation study suggest that in practice, SSA may be a reliable tool to detect frequent adverse drug reactions when medicine uptake trends are variable. Use of SSA as a signal detection tool will help to ensure that adverse events associated with medicines are identified early and excess harm avoided. In the scenarios considered in this study, adjustment for underlying medicine utilization trends in a newly market medicine effectively overcomes under-ascertainment bias. The method identified an increased risk of one medicine, indicative of an adverse event, being prescribed after another in scenarios where a true association existed. These results suggest that SSA may be effectively applied as a safety signal detection tool for newly marketed medicines where sufficiently large health claim data are available.

## Competing interests

The authors have no conflict of interest. Dr. Pratt is funded by the National Health and Medicine Research Council Early Career Fellowship.

## Authors’ contributions

NP conceived and designed the study, performed the statistical analysis and prepared the first draft of the manuscript. JI participated in the design and statistical analysis of the study and helped draft the manuscript. CR participated in the design of the study and helped to draft the manuscript. ER participated in the design of the study and helped to draft the manuscript. All authors read and approved the final manuscript.

## Pre-publication history

The pre-publication history for this paper can be accessed here:

http://www.biomedcentral.com/1471-2288/14/66/prepub
